# Karawun: a software package for assisting evaluation of advances in multimodal imaging for neurosurgical planning and intraoperative neuronavigation

**DOI:** 10.1007/s11548-022-02736-7

**Published:** 2022-09-07

**Authors:** Richard Beare, Bonnie Alexander, Aaron Warren, Michael Kean, Marc Seal, Alison Wray, Wirginia Maixner, Joseph Yuan-Mou Yang

**Affiliations:** 1 Developmental Imaging, Murdoch Children’s Research Institute, Melbourne, Australia; 2 Department of Neurosurgery, Neuroscience Advanced Clinical Imaging Service (NACIS), The Royal Children’s Hospital, Melbourne, Australia; 3 Neuroscience Research, Murdoch Children’s Research Institute, Melbourne, Australia; 4 Medical Imaging, The Royal Children’s Hospital, Melbourne, Australia; 5 Department of Paediatrics, The University of Melbourne, Melbourne, Australia; 6 National Centre for Healthy Ageing, Melbourne, Australia; 7 Department of Medicine (Austin Health), The University of Melbourne, Melbourne, Australia; 8 The Florey Institute of Neuroscience and Mental Health, Melbourne, Australia; 9 Peninsula Clinical School, Central Clinical School, Monash University, Frankston, Australia

**Keywords:** Image-guided surgery, Tractography, Diffusion imaging, DICOM

## Abstract

**Purpose:**

The neuroimaging research community—which includes a broad range of scientific, medical, statistical, and engineering disciplines—has developed many tools to advance our knowledge of brain structure, function, development, aging, and disease. Past research efforts have clearly shaped clinical practice. However, translation of new methodologies into clinical practice is challenging. Anything that can reduce these barriers has the potential to improve the rate at which research outcomes can contribute to clinical practice.

In this article, we introduce *Karawun*, a file format conversion tool, that has become a key part of our work in translating advances in diffusion imaging acquisition and analysis into neurosurgical practice at our institution.

**Methods:**

*Karawun* links analysis workflows created using open-source neuroimaging software, to Brainlab (Brainlab AG, Munich, Germany), a commercially available surgical planning and navigation suite. *Karawun* achieves this using DICOM standards supporting representation of 3D structures, including tractography streamlines, and thus offers far more than traditional screenshot or color overlay approaches.

**Results:**

We show that neurosurgical planning data, created from multimodal imaging data using analysis methods implemented in open-source research software, can be imported into Brainlab. The datasets can be manipulated as if they were created by Brainlab, including 3D visualizations of white matter tracts and other objects.

**Conclusion:**

Clinicians can explore and interact with the results of research neuroimaging pipelines using familiar tools within their standard clinical workflow, understand the impact of the new methods on their practice and provide feedback to methods developers. This capability has been important to the translation of advanced analysis techniques into practice at our institution.

**Supplementary Information:**

The online version contains supplementary material available at 10.1007/s11548-022-02736-7.

## Introduction

How can a busy clinician identify and test neuroimaging research outcomes that have potential to improve patient care? Conversely, how can a researcher in a technical discipline investigate the potential impact of their work on clinical practice? Communication between technical disciplines and clinical practitioners can be difficult due to very different educational backgrounds and nuances unique to each field.

In this paper, we introduce *Karawun*,^1^ a software tool, that we have found useful in the context of neurosurgery. *Karawun* converts the results of research diffusion-weighted imaging (DWI) and tractography pipelines to a form that can be imported into a widely used clinical software package, Brainlab CX(Brainlab AG, Munich, Germany). Neurosurgeons can then explore and interact with the data produced by researchers and do so in a familiar clinical environment. This allows a neurosurgeon to compare pipeline outputs and address questions, such as “does a method offer me new insights?”, “Does it work better on some patients than others?”, and “Does a recently published enhancement perform even better?” It also highlights the evidence-practice gap to clinicians, allowing them to provide feedback to vendors about new methods they find useful.

### Overview of imaging in neurosurgical planning and navigation

MR-based imaging techniques are important in presurgical planning and intraoperative guidance for modern neurosurgical practice [1, 2]. Critical information may be derived from routinely acquired clinical MRI sequences as well as advanced neuroimaging research techniques that have clinical potential.

When performing neurosurgery in or in proximity to eloquent brain regions, advanced neuroimaging techniques, such as task-based blood-oxygenation level-dependent functional MRI (BOLD-fMRI) and diffusion MRI tractography are utilized to locate eloquent cortex and the associated subcortical white matter (WM) tracts [3–7]. Accurate delineation of these eloquent brain structures against the proposed lesion resection is critical to avoid surgical injuries, and to ensure postoperative functional preservation. In epilepsy brain surgeries, multimodal structural and functional MRI data are utilized to help define the epileptogenic zone together with clinical electrophysiological data, and to locate the proposed epileptogenic lesions, such as focal cortical dysplasia [1, 8]. Examples include, but are not limited to, the combination of different structural MRI sequences (e.g., T1-weighted, T2-weighted and fluid-attenuated inversion recovery (FLAIR) imaging), and cerebral metabolic imaging of both the seizure (ictal) and between seizure (inter-ictal) states (e.g., the inter-ictal ^18^F-fluorodeoxyglucose positron emission tomography (^18^F-FDG-PET), and the ictal single-photon emission computerized tomography (SPECT)). Combined outputs of both the conventional clinical MRI data and various post-processed images derived from advanced neuroimaging data may assist presurgical planning in, for example, determining the resection target, surgical positioning of the patient, and deciding the most appropriate surgical corridor. During the surgery, this imaging information may be used to complement and to guide functional brain mapping via direct cortical and subcortical electrostimulation [3, 9, 10], and to assist with formulation of the intracranial electrode implantation strategy, ensuring accurate and adequate electrode coverages, as in MRI-negative epilepsy surgery cases to help confirm the seizure-onset zone [11].

### Advances in diffusion-weighted imaging with potential clinical application

Although not yet utilized in clinical neurosurgical practice, methods of acquiring and processing diffusion MRI data, and tractography reconstructions have substantially advanced due to significant research efforts over the past two decades [12–15]. Every step involved in generating the tract images has benefited: from acquisition to DWI data preprocessing, WM microstructural modeling and tractography reconstruction. Advances in MRI hardware and sequences now allow DWI data appropriate for advanced modeling to be acquired in clinically feasible times [16]. Work on improved tractography has focused on overcoming the limitations of the classical diffusion tensor imaging (DTI) modeling approach [17] by developing models allowing estimation of multiple fiber orientations per MRI voxel and tracking algorithms that thoroughly explore the possible range of WM tract configurations [18–21]. This facilitates more anatomically accurate tractography reconstructions. Many of these methods are implemented in open-source and/or freely available software packages and are associated with active communities of developers and users [22].

### Barriers to clinical translation of advanced imaging techniques

The wide range of neuroimaging software tools created by the research community represents an enormously powerful and flexible resource that can be used to develop and test new MRI acquisition and processing approaches. However, the clinical uptake of advanced neuroimaging methods implemented by these tools is very limited [23]. It is hard for clinicians to evaluate the improvements to clinical care that they may offer. The barriers to clinicians using the tools directly are high. A “clinical application” built using research software components is usually quite complex. For example, a DWI software pipeline used to generate a tract reconstruction involves many steps. Each of these steps may be performed using several different approaches that have been developed by the research community and be appropriate in different circumstances and a different software component may implement each option. Slightly different combinations may be appropriate in different circumstances. Creating, using, and modifying complete imaging pipelines is a specialized activity, requiring a significant investment of time and effort, and understanding of the nuances of file formats and various programming interfaces. Tracking and evaluating the latest research outputs requires dedication of resources over a long period of time.

Translation of new image acquisition and processing methods from the research domain to clinical practice is critically important in the pursuit of improved care for neurosurgical patients. Image acquisition and analysis are very active areas of research and innovations made by researchers have the potential to enhance neurosurgical planning and navigation and therefore contribute to improvements in patient care, especially for complex cases. However, neurosurgeons and vendors face a “chicken and egg” problem in that it is difficult to evaluate new imaging methods in a clinical environment, but it is impractical for vendors to provide new methods without recommendations from clinicians. It may also be dangerous to rely on commercially available tools when limitations of algorithms they offer are well known [24]. The problem is compounded by the range of imaging research that is potentially relevant to neurosurgical practice and the complex regulatory, technical and clinical environment in which commercial vendors must operate.

### Reducing translation barriers with Karawun

We have found that providing results of analysis using research software to neurosurgeons via Brainlab, the surgical planning and navigation platform used in our institution, reduces translation barriers in several ways. It allows neurosurgeons to interact with the analysis results in a familiar neuronavigation environment, thus eliminating the need to understand the mechanics of generating the analysis and the learning curve associated with a piece of research visualization software. Converting the analysis results to a form that matches the internal Brainlab structure leads to a richer and more cohesive experience than is possible via screenshots—tracts and tumors are represented as 3D objects, as if they were created in Brainlab, and can be used to conduct detailed surgical planning and be made available during surgery. Neurosurgeons are thus able to compare the results of different analysis choices on surgical decisions and provide feedback to researchers and vendors.

## Overview of Karawun

### Functionality

**Fig. 1:**
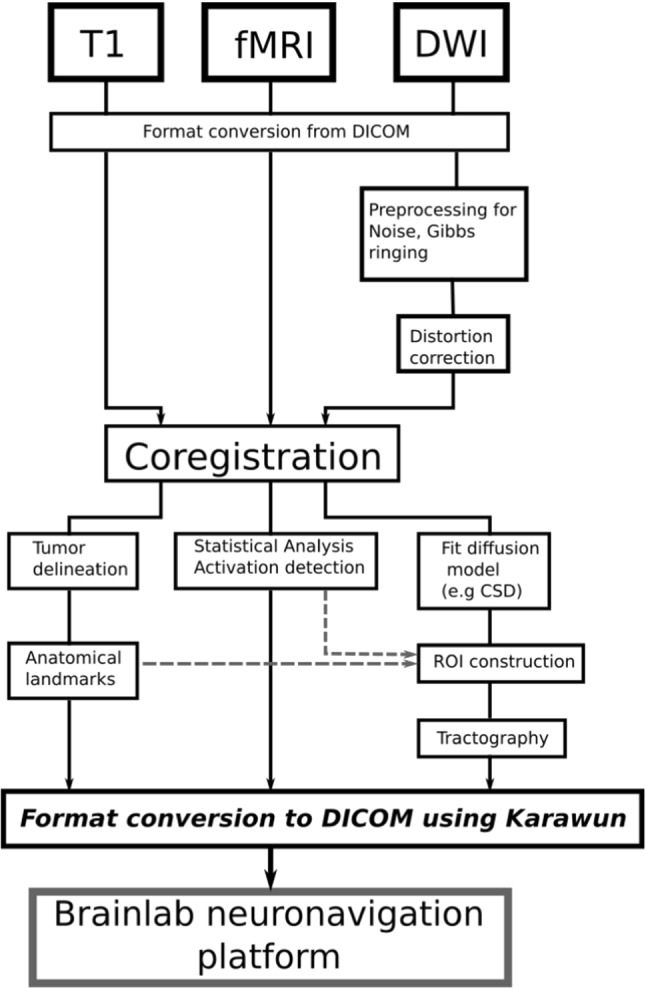
A typical neuroimaging workflow for neurosurgical planning using neuroimaging research software

*Karawun* is a python package with a single command-line interface (see Example). It is available for Linux, OSX, and Windows. Figure 1 illustrates a typical use case. It converts the results of a neurosurgical workup, created using open-source research tools, to a Digital Imaging and COmmunications in Medicine (DICOM) format compatible with Brainlab. A neurosurgical workup will typically consist of one or more single component, coregistered NIfTI images (e.g., T1 weighted, Gd contrast, FLAIR, FA map), one or more MRtrix3 [22] format tck files and optionally one or more coregistered NIfTI mask or label images, representing structures, pathology or functional activation. NIfTI images are represented using the standard DICOM MR modality and assigned to a common frame of reference. Mask or label images and tck files are represented using Segmentation Information Object Definition (IOD) (SEG modality (tag 0008,0060)). Streamlines in tck files are represented using the Line Sequence surface mesh primitive of the Surface Mesh Module in the Surface Information Entity (IE). Mask or label images support 30 labels, each mapped to a distinct color and stored using run length encoding described in Sect. 8.2.2 and Annex G (https://dicom.nema.org/medical/Dicom/2017c/output/chtml/part05/sect_8.2.2.html, https://dicom.nema.org/medical/Dicom/2017c/output/chtml/part05/chapter_G.html).

Conversion utilizes a “template” DICOM file, typically from the session being analyzed, to provide patient and institutional tags.

Vendor support for quantitative DICOM, including SEG modality, is improving and makes transfer between research pipelines and commercial software possible [25, 26].

### What Karawun is not

*Karawun* is not a DICOM reader. It does not compete with or attempt to duplicate the functionality of the many tools that convert DICOM into NIfTI as the first step in neuroimaging data analysis [27]. Issues such as gradient directions in DWI data and interaction with patient orientation are dealt with by those tools so that the following pipelines do not have to.

*Karawun* creates a small set of possible DICOM modalities that are useful for the neurosurgical workup use case, i.e., it cannot create all possible DICOM output formats. It is not possible, for example, to perform a distortion correction on a NIfTI diffusion dataset and convert the result to a DICOM form that would allow analysis by Brainlab. *Karawun* was developed and tested for use with Brainlab.

## Example—comparing modeling and tractography methods on a clinical case

### Overview

The example illustrates how neurosurgeons may investigate the advantages offered by state-of-the-art acquisitions, diffusion modeling, and tractography. Two workups were created using open-source research tools (See Supplementary Sect. 4 for details). One used traditional DTI modeling and deterministic tractography, approximating the capabilities that were, until very recently, offered by commercial vendors. The second used a more complex pipeline, starting with a multi-shell, high b-value acquisition, using multi-tissue CSD WM modeling and probabilistic tractography [22, 28]. Distortion correction using reverse phase encoding was included in both pipelines. The workup also included a motor BOLD-fMRI task and manual delineation of a tumor, both performed using standard neuroimaging research tools.

The purpose of this example is not to advocate for one processing pipeline or family of tools, but to illustrate that the results of complex tractography workups, the equivalent of which are difficult or impossible to duplicate using commercial tools, can be presented to neurosurgeons in a familiar visualization, planning and navigation suite.

### Participant

A child aged between 10 and 15 presented with a large left parieto-occipital brain tumor revealed on diagnostic MRI. No language, motor, or visual deficits were detected on formal neurological and functional examinations. An open craniotomy and surgical debulk of this tumor were performed, followed by cranial irradiation therapy and chemotherapy. The right-hand motor functional cortex, left corticospinal tract and the left optic radiation were mapped to establish a safe surgical corridor and trajectory.

### Conversion with Karawun

Karawun provides a single command-line utility, importTractography, to perform conversion of NIfTI and tck files to DICOM. Conversion for Brainlab was performed using the following command:

--dicom-template: a DICOM file used to provide institutional and patient information. Selected tags are copied from this file to create the new DICOM files. Typically, one of the DICOM files used to create the inputs to the processing pipeline would be used for this purpose. In this example, the DICOM template file was used to create the T1 NIfTI image from which T1brain.nii.gz was derived.
--nifti: Typically structural images in NIfTI format that will be converted to MR modality DICOM. Multiple component images, such as fMRI time series or raw diffusion data, are not supported. All arguments are assumed to be coregistered and are assigned to a common frame of reference in the output DICOM dataset. *Karawun* does not perform or check coregistration.
--tract-files: Streamline files created by MRtrix3. The converted forms are marked as deriving from the first image listed in the --nifti option arguments and therefore will be associated with that image in Brainlab until the frame of reference is accepted in the ImageFusion module.
--label-files: NIfTI images containing masks or regions of interest. Each image may have up to 30 distinct integer values, each representing a different region of interest, which will be displayed in different colors in the converted form. The voxel size, spacing, and orientation of each argument must match one of the images listed in the --nifti option, and the output DICOM will be marked as deriving from that image.
--output-dir: The name of the target folder (which will be created if it doesn’t exist) containing the output DICOM, ready for import into Brainlab.

## Results

**Fig. 2:**
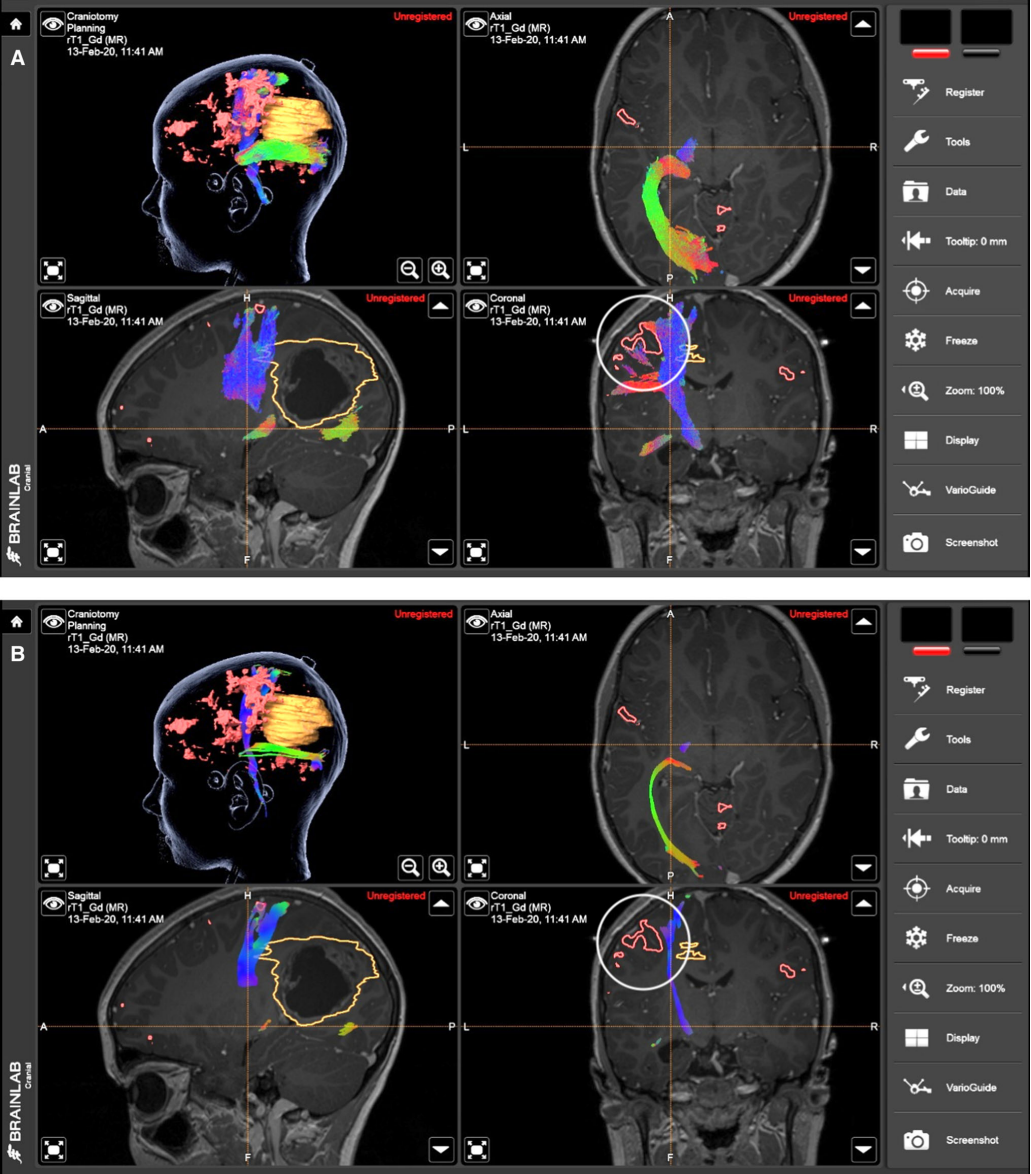
Display of the neurosurgical workup using CSD and probabilistic tractography (panel A) and DTI and FACT tractography (panel B). Display is with the Brainlab Cranial Navigation software. The tumor is rendered in yellow and fMRI motor activation in red

The Brainlab Cranial Navigation platform view of the clinical case example is shown in Fig. 2. The two panels demonstrate streamline objects representing the left corticospinal tract and the left optic radiation, colored by streamline orientation, and segmentation objects displaying the tumor (yellow) and areas of BOLD-fMRI motor activation (red). These objects are overlaid on the coregistered T1-weighted image in this example, although may be overlaid on any other scans that were imported. The tumor and BOLD-fMRI activation are visible as solid objects in the 3D view, and as outlines in the slice views.

The neuronavigation software allows clinicians to explore important spatial relationships between the tumor, the adjacent WM tracts, areas of peri-tumoral motor BOLD-fMRI activation, and anatomical features visible in the background MRI.

Panel A shows a Brainlab visualization of tractography reconstructed using a method combining the multi-tissue CSD model and a probabilistic tracking algorithm, available in MRtrix3. Panel B shows the same tracts reconstructed using DTI modeling and Fiber Assignment by Continuous Tracking (FACT) tracking algorithm [29]. Larger gaps between the tumor margins and both WM tracts are visible in Panel B than A. In Panel B, there is a failure to reconstruct the lateral face-motor projections of the corticospinal tract and lack of spatial overlap between activated finger-motor cortex and the tract, as indicated by the white circles. Inadvertent injuries to these WM tracts due to such false-negative tracking problems will likely result in postoperative permanent motor and/or visual field deficits. For this patient, the resection margins were informed by multi-tissue CSD-based tract objects (i.e., images from Panel A) and there were no transient or permanent functional deficits following surgery.

An additional example using data distributed with the source code is provided in Supplementary Sect. 5.

## Discussion

We have introduced *Karawun*, a tool for conversion of neurosurgical workups created using research software—NIfTI images and tck tractography files—to DICOM datasets compatible with the Brainlab surgical planning and navigation suite. We will continue to develop *Karawun* as vendor support for DICOM standards evolves, allowing the output to be vendor independent. Our work to date demonstrates the benefit of linking research workflows to commercial neurosurgical navigation systems. We have tested this concept using Brainlab as the demonstration platform and anticipate that the same strategies will be feasible with other commercial products as support for DICOM standards is improved.

*Karawun* has been created for the purposes of research and is distributed as is, without warranty, as is the common practice for most research software. The medico-legal issues associated with combining clinical practice and research results are complex, vary with legal jurisdiction and must be judged on a case-by-case basis. Our aim is to allow clinicians to develop an understanding of the benefits of research developments and provide feedback to vendors. As a result, vendors will therefore be in a position to assess demand for new approaches, assist with formal evaluation of the benefits, and develop products that meet complex regulatory requirements.

*Karawun’s* role is at the output end of the analysis pipeline, and its potential importance is best illustrated by briefly describing the progress of translating advanced neuroimaging into pediatric neurosurgical practice at our institution over the last 10 years. Early work, conducted by JYMY as a PhD student and advanced neurosurgical trainee, involved applying research diffusion modeling and tractography (CSD and probabilistic tractography) to High angular resolution diffusion imaging (HARDI) sequences acquired as part of routine care of patients in the neurosurgery epilepsy program [7, 30, 31]. Advances made by the team during this period included the ability to acquire a HARDI sequence in clinically feasible times and an understanding of how to build analysis pipelines addressing neurosurgical questions. Results were presented to the multidisciplinary clinical team for presurgical planning, in the form of screenshots, collated in PowerPoint, or flythrough videos created using ParaView [32]. Screenshots were made available on both PC workstation and wall mount TV screens in the operating room. Screenshots provide a useful but limited window to complex surgical planning data—they capture a set of pre-defined views, but no further interaction is possible during surgery. For example, new views cannot be created by rotating the data, altering the viewpoint, or toggling the visibility of objects. These limitations led us to identify a clinical demand for a means to incorporate the planning data in the surgical planning and guidance platforms. We developed *Karawun* for use with Brainlab. There may be interest in using the results with other surgical navigation systems, specifically the StealthStation family of products by Medtronic Inc, Minneapolis, Minnesota. The version available when *Karawun* was developed, StealthStation S7, was not able to display 3D streamlines and had no mechanism for importing segmentation objects. Thus, DICOM files containing streamlines and segmentation objects cannot be imported into S7 but the standard MR modality files generated by *Karawun* can. It is important to note that alternatives are available for StealthStation S7 as it can directly import NIfTI files and can transform them into segmentation objects via thresholding, etc. following importation. The recently released StealthStation S8 and Stealth Tractography modules do support display of 3D streamlines. *Karawun* has not yet been tested with this platform.

Karawun’s conversion to an interchange format is not the only way of achieving integration between commercial surgical planning and navigation suites and external systems. OpenIGTLink facilitates a real-time link between suites, such as Brainlab, and external software, including 3D Slicer and mrview [33, 34].

Limitations of the commercial tools were clearly apparent during this period. The only tractography choice available was a combination of the DTI model and a FACT tracking algorithm available in Brainlab. The HARDI acquisitions were not recognized by the import process and no probabilistic tracking algorithms were available. The situation changed significantly following the local deployment of *Karawun* in early 2018. A team of four neuroimaging specialists now routinely conduct neurosurgical imaging workups, using neuroimaging research software, for epilepsy and selected brain tumor and vascular malformation patients, involving language and motor fMRI, structural MRI, DWI and tractography, cortical venous mapping and inter-ictal ^18^F-FDG-PET, ictal SPECT maps. Workups of complex surgery cases are converted using *Karawun* and made available to the surgeons for planning and navigation purposes. Our experience to date demonstrates that *Karawun* has made a transformative impact in a range of difficult surgical scenarios. In addition to the traditional problem of mapping of eloquent cortex and related subcortical WM tract position with high risk of surgical injury, it has also allowed neurosurgeons to establish safe surgical corridors and resection trajectories and define safe resection margins. *Karawun* was also helpful in other difficult surgical scenarios, including: (i) to display the co-localized epileptogenic zone, as proposed by the preoperative multimodal imaging-based workup, that enabled accurate intracranial electrode implantation during the first stage of two-stage epilepsy surgeries. The implanted electrodes were used for recording and monitoring electrophysiology of the proposed epileptogenic zone (thus to affirm or to adjust the resection plan), and to enable extraoperative language and motor brain mapping by electrostimulating the implanted electrodes that further validated the localization information proposed by the fMRI and tractography data, prior to explantation and definitive resective surgery performed one week following electrode implantation. (ii) for stereotactic re-insertion of an Ommaya catheter into a cystic low-grade thalamic brain tumor, whereby the catheter trajectory was near both the corticospinal tract fibers at the posterior limb of the internal capsule, and the optic radiation fibers at the retrolenticular portion of the internal capsule.

The team processed 16 and 23 cases of such complex surgeries in years 2020 and 2021, respectively. This represents all 39 supratentorial resective epilepsy and selected non-urgent brain tumor cases performed in the eloquent brain regions in the last two years. This includes 13/36 (36%) and 17/31 (55%) resective epilepsy surgery cases performed in years 2020, and 2021, respectively. The increase in case proportion from one year to next is explained by the availability of *Karawun* and our team’s growing experience enabling our program to take up more surgically challenging cases.

Specific examples of high-risk surgery illustrating our clinical learning curve with *Karawun* include our previously reported one-stage, language dominant, insular-opercular epilepsy surgery series, and the related bottom-of-sulcus-dysplasia (BOSD) epilepsy surgery series, representing over 10 years of our clinical experience, performed with advanced fMRI and tractography guidance [35, 36]. We demonstrated excellent surgical and seizure outcomes in both surgical series, without the need to perform additional invasive procedures for functional brain mapping, including in an awake craniotomy setting, which is unsuited for the majority of children. The type of advanced neuroimaging-assisted surgical workup described above was employed in the majority of the cases reported in both series, with the later cases all being guided by the use of *Karawun* when it became available in early 2018.

Thus, the ability to make data available to clinical end users in their clinical workflows has had a transformative impact in clinical neurosurgical practice at our institution and has been invaluable in our efforts to improve patient care using advanced diffusion MRI tractography processing methods. We hope that others will find it similarly useful.

## Supplementary Information

Below is the link to the electronic supplementary material.

Supplementary file1 (DOCX 2262 KB)

## Data Availability

Source code, including test data, is available on GitHub at https://github.com/DevelopmentalImagingMCRI/karawun. Documentation is available at https://developmentalimagingmcri.github.io/karawun.Details of installation, testing, and validation procedures and importing to Brainlab are available in Supplementary material.

## Footnotes

1 Karawun is a grass used as a fiber crop by Aboriginal people in southeastern Australia.
