# Supplementary material for: Karawun: a software package for assisting evaluation of advances in multimodal imaging for neurosurgical planning and intraoperative neuronavigation
Source: Int J Comput Assist Radiol Surg. 2022 Sep 7;18(1):171–9. doi: 10.1007/s11548-022-02736-7 (PMC9883338; doi:10.1007/s11548-022-02736-7)
Supplement: Supplementary file 1 — Supplementary file1 (DOCX 2262 KB) [file 11548_2022_2736_MOESM1_ESM.docx]

Supplementary Material

# Installation

Karawun is distributed via the Python Package index and conda-forge - see online instructions at https://developmentalimagingmcri.github.io/karawun/installation.html.

# Dependencies

*Karawun* uses pydicom to provide all DICOM reading (template) and writing operations[8]. Geometric computations and reading and slicing of NIfTI files use SimpleITK[9–11].

# Continuous Integration testing and validation

*Karawun* is published from a protected branch of the github repository, with pull requests required to pass automated continuous integration (CI) regression tests and approval prior to merging. The tests cover a matrix of python versions (3.6, 3.7, 3.8, 3.9) and platforms (Linux, OSX and Windows) and verify that all output files are identical to those at baseline, with python mocking used to force timestamps and unique identifiers to baseline values.

Two sets of test data are available to allow users to validate their own installations - the anonymized data from a healthy volunteer provided for regression testing and available in the source code repository and a second repository of synthetic data (https://github.com/DevelopmentalImagingMCRI/karawun_test_data.git), derived from templates distributed with FSL[12], to confirm that aligned images with different data stride patterns and voxel dimensions remain aligned with each other, the label objects and synthetic streamlines after conversion to DICOM and importation to Brainlab. Conversion of the anonymized data is described in Section 5.

# Image acquisition and processing for clinical case.

## MRI acquisition

Presurgical MRI was performed on a 3Tesla Siemens MAGNETOM Prisma scanner with a 64-channel head coil receiver. The following sequences were acquired: volumetric T1-weighted with and without Gadolinium contrast, T2-weighted and non-volumetric FLAIR, multi-shell DWI (b=3000/2000/1000 s/mm2, 60/45/25 directions) and a right index finger tapping task-based motor BOLD-fMRI scan (standard block design paradigm: 15 secs active finger-tapping blocks, alternating with 15 secs rest blocks, 195 secs in total).

## Image processing

### Coregistration

The T1-weighted images were linearly coregistered to the distortion corrected b0 volume of the DWI data using FMRIB Linear Registration Tool (FLIRT) from FSL (version 6, FMRIB's Software Library; [www.fmrib.ox.ac.uk/fsl](http://www.fmrib.ox.ac.uk/fsl)). Probabilistic multi-tissue CSD and deterministic DTI tractography processing

All DWI processing and tractography reconstruction were performed using MRtrix3 [1].

A multi-tissue CSD technique was used to estimate tissue response function and to model WM fiber orientations [2]. Tractography was performed using an iFOD2 probabilistic tracking algorithm [1]. Corticospinal tract and optic radiation tractography were reconstructed based on our previously published methods [3, 4].

An additional pair of the same corticospinal tract and optic radiation was generated for comparison purposes using the same sets of tracking ROIs, and using a DTI model estimated from the b=1000 mm/s^2^ shell of the multi-shell DWI data, and tractography using the FACT algorithm implemented in MRtrix3 [5, 6]. The DTI/FACT combination is the same tractography tool originally available in Brainlab.

### FMRI processing

A standard block design analysis of the motor finger-tapping task was performed using FSL-FEAT (FMRI Expert Analysis Tool) Version 6.00 [7]. The processing steps included: non-brain removal, corrections for slice-timing motion, b0 unwarping and registration to T1 using FLIRT, automated fMRI data noise removal (ICA-AROMA), spatial smoothing, and high-pass temporal filtering. Time-series statistical analysis was carried out using FILM with local autocorrelation correction. Z statistic images were thresholded using clusters determined by Z > 3.1 and a cluster significance threshold of P = 0.05, corrected for multiple comparison.

### Tumor delineation

A tumor mask was delineated manually using the mrview visualization tool.

# Example - Healthy volunteer - Corticospinal tracts and text labels

The GitHub repository includes anonymized data from a healthy volunteer that is used as part of the CI testing and is useful to confirm operation of an installation of *Karawun*. This example will illustrate the process of viewing the results of an analysis in the research environment, then converting to DICOM and importing into and viewing in Brainlab. Linux/OSX command line syntax will be used in the descriptions.

## Ethics Statement

A healthy volunteer was scanned during the pilot phase of a project that received ethical approval from The Royal Children's Hospital Melbourne Human Research Ethics Committee (HREC). The volunteer gave informed, written, consent to publicly release an anonymized version of the MR data for distribution with *Karawun*.

## Analysis results

The analysis results we will explore are located in karawun/tests/Data/Tractography in the source code repository and consist of a scalped T1 weighted image (T1brain.nii.gz), a coregistered FLAIR image, a label image containing some 3D text saying “Karawun” (words.nii.gz) and the left and right corticospinal tracts (Left_PT_final.tck and Right_PT_final.tck). This data can be viewed in the mrview visualization tool available in MRtrix3 (Figure 1).


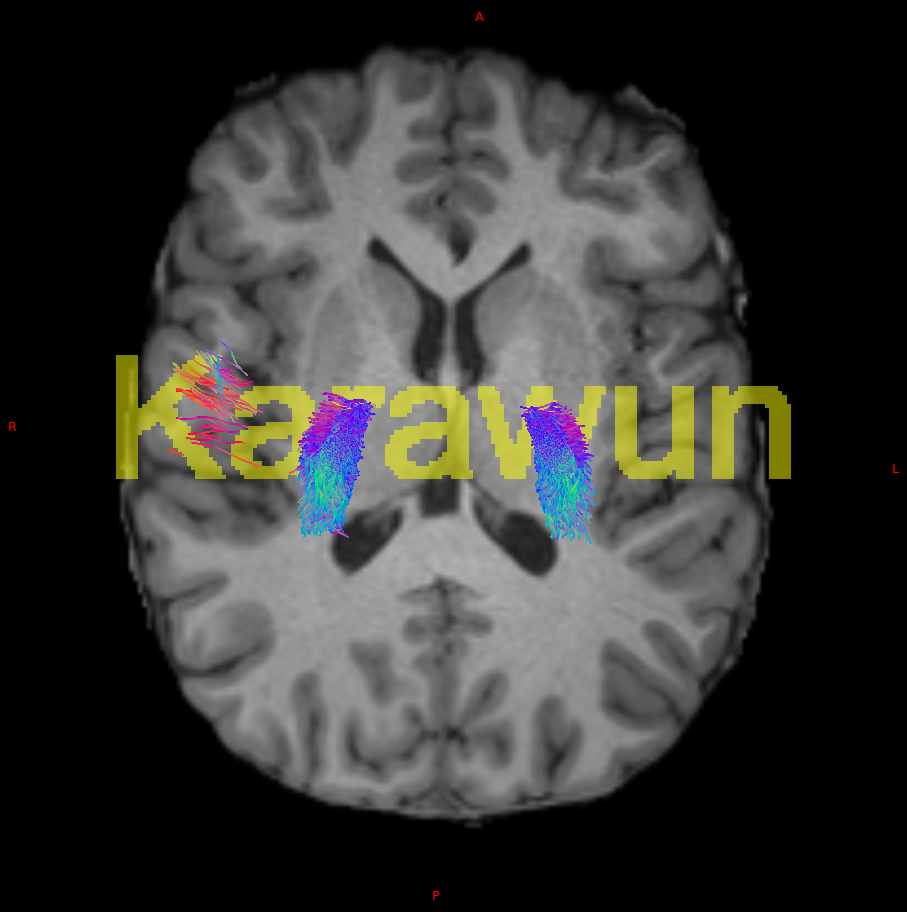

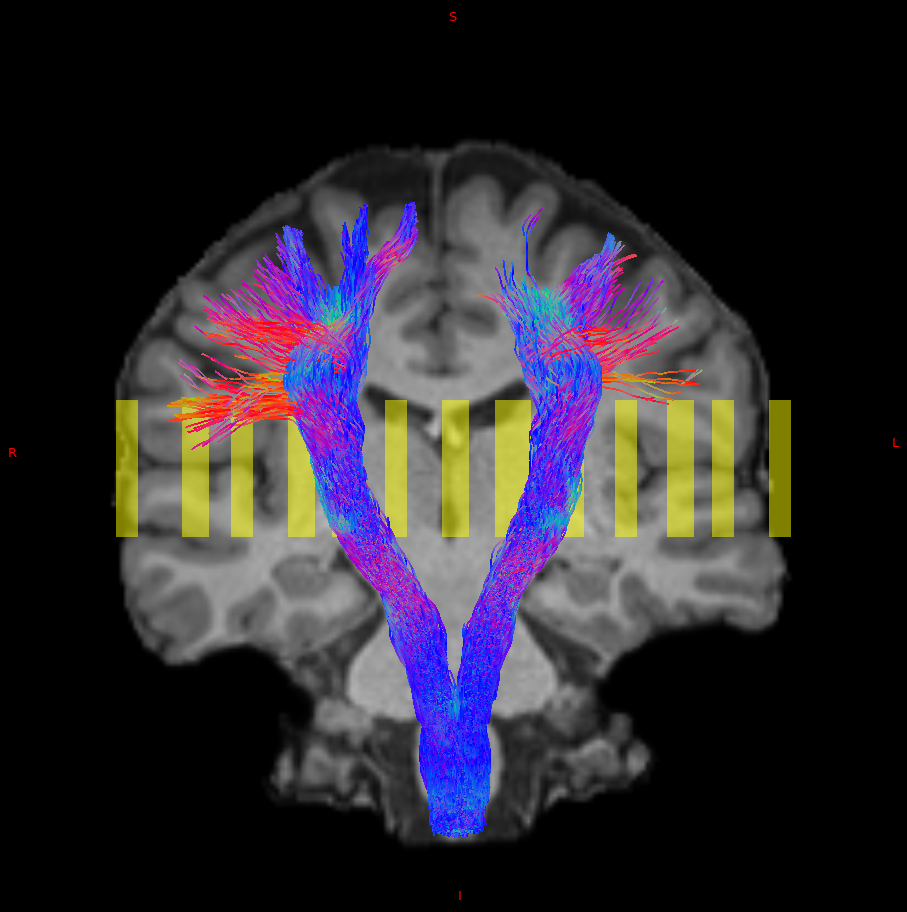


Figure 1: Tractography and mask data for a healthy volunteer viewed in mrview.

## Conversion to DICOM

Karawun provides a single command-line utility, importTractography, to perform conversion of NIfTI and tck files to DICOM. The following command, executed from the karawun source code repository, will perform the conversion:

importTractography --dicom-template tests/Data/Dicom/1.3.12.2.1107.5.2.43.167031.2019040213095021814319052.dcm \

--nifti tests/Data/Tractography/T1brain.nii.gz tests/Data/Tractography/FLAIRbrain.nii.gz \

--tract-files tests/Data/Tractography/Left_PT_final.tck \

tests/Data/Tractography/Right_PT_final.tck \

--label-files tests/Data/Tractography/words.nii.gz \

--output-dir ForBrainlab

This command creates the ForBrainlab folder containing the following subfolders:

FLAIRbrain/

Left_PT_final/

Right_PT_final/

T1brain/

words/

Each of which contains a DICOM series.

## Visualisation in Brainlab

The entire folder hierarchy can be imported into Brainlab via the Originserver interface (see <https://developmentalimagingmcri.github.io/karawun/importing.html>). The patient name in this anonymized dataset is “DevImConvTest” and the study description is “TestStudy”. Both are copied from the --dicom-template argument. The Series Description of each series matches the containing folder name, derived from the NIfTI image or track filename. The frame of reference needs to be accepted in Brainlab’s ImageFusion module before all data can be combined.

Figure 2 illustrates the Brainlab view of the converted data. Panels A and B show traditional axial and coronal views of the 3D datasets with different structural images in the background. The segmentation objects, i.e. the streamlines and letters, have been colored, sliced and overlaid on the background image. A 3D rendering produced by Brainlab is shown in Panel C. Brainlab is capable of a range of visualization modes combining volume and 3D surface data, including cropping and volume rendering in addition to rotation of and flying through the scene. Here we crop the T1 volume in the coronal plane to expose the rendered 3D objects. This view shows that the imported segmentation data is three-dimensional, rather than a series of 2D color overlays. Streamlines in the cropped zone are “floating” above the letters. The data can also be used directly in Brainlab planning and navigation modules, such as Cranial Navigation.


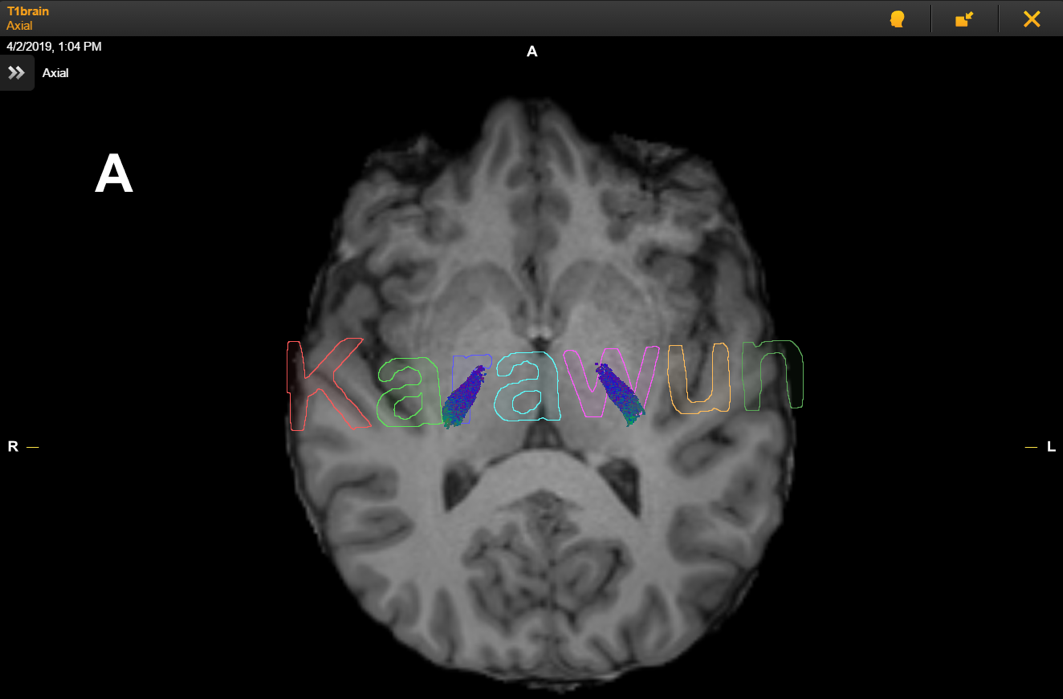


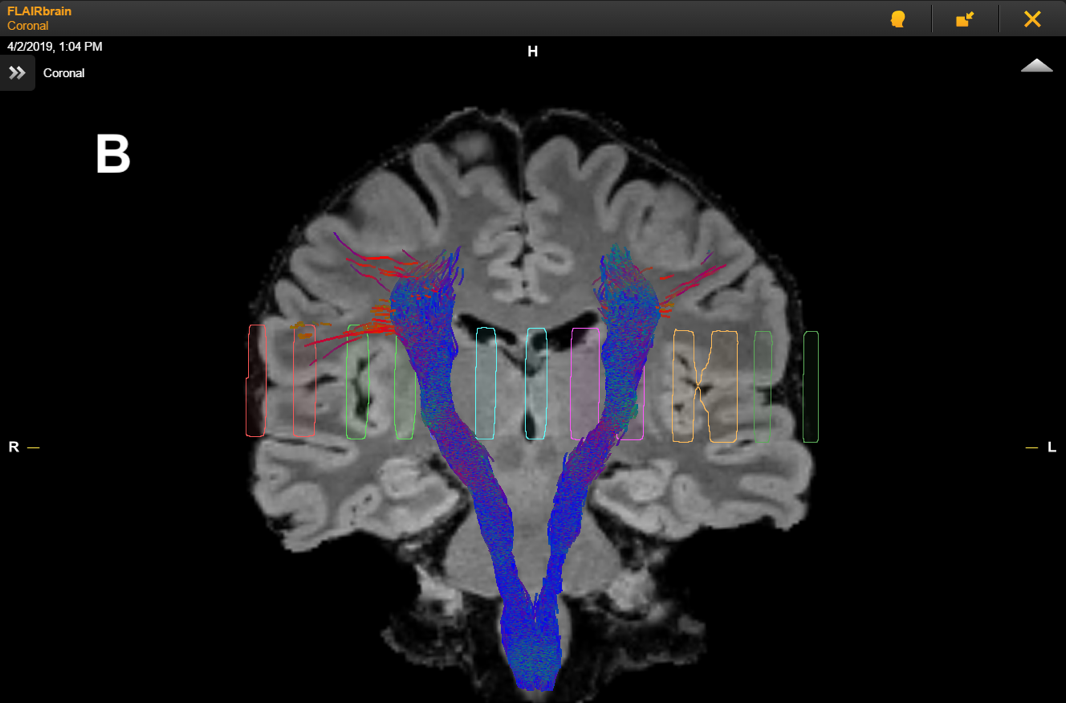


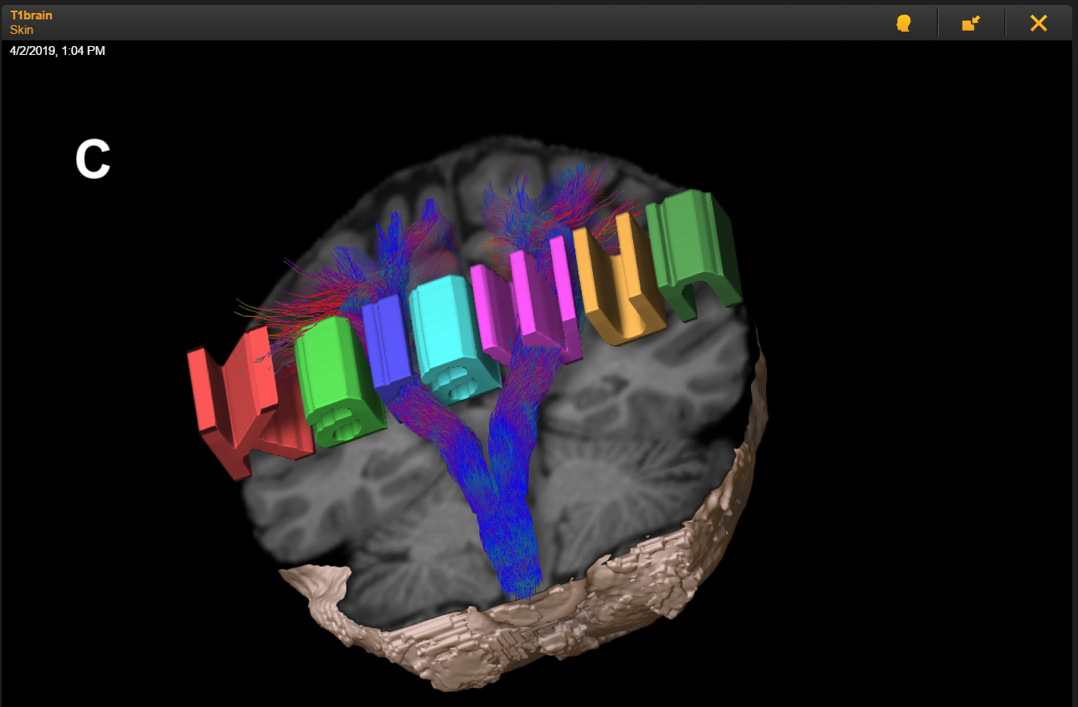


Figure 2: Display of converted data in Brainlab. Panel A illustrates an axial view of the T2 volume with the 3D objects overlaid. Outlines of the letters are visible and a slice through the 3D streamlines is visible. Panel B illustrates a coronal view of a coregistered FLAIR scan with the same 3D objects. Panel C illustrates Brainlab’s 3D rendering of the same data, with a coronal cropping. The letter and streamline objects are not sliced in this mode and their 3D representation is clearly visible, with streamlines “floating” among the letters.

# Validation Dataset

It is essential that geometry and orientation of data is preserved by the conversion process, irrespective of the structure of the original NIfTI data. It is recommended that scenes viewed in Brainlab are compared to those viewed in the packages used to perform the analysis, in order to uncover any unexpected behavior. We have created a synthetic dataset (https://github.com/DevelopmentalImagingMCRI/karawun_test_data) to help provide assurance that the conversion process is robust to variation in NIfTI source data.

The dataset is derived from the FSL template brain with left/right markings [12]. It includes label versions of the letters and synthetic streamlines that mimic the L/R markings. There are multiple versions of the raw data and the label images, with different data stride patterns, and with different slice thicknesses. Conversion of all data using *Karawun* allows alignment between data with different NIfTI formats to be verified.

Conversion can be performed using the following command from inside the Images folder:

importTractography -d ../Dicom/1.3.12.2.1107.5.2.43.167031.2019040213095021814319052.dcm --nifti MNI152_T1_2mm_LR-masked.nii.gz mni_* --label-files LR* --tract-files letters.tck -o Validation

Figures 3 and 4 indicate the expected output. There are 21 variants of stride and slicing patterns. The expected behavior is for Brainlab to display all data in the same space (i.e. perfectly aligned aside from resampling noise).


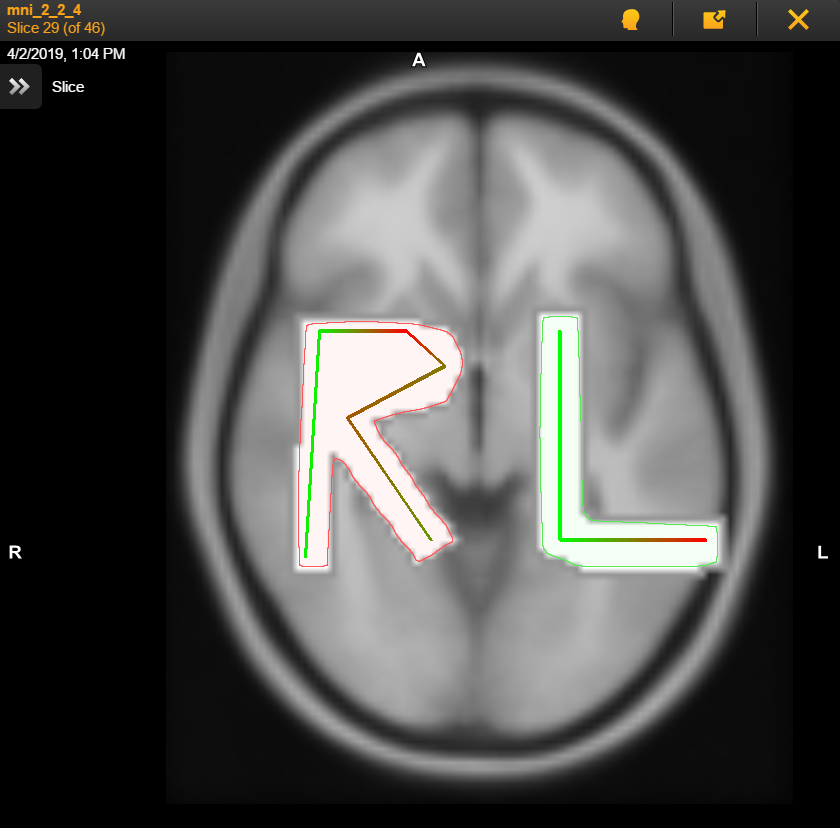


Figure 3: Brainlab axial view of imported template MRI, R/L masks and R/L letters constructed from synthetic streamlines overlaid.


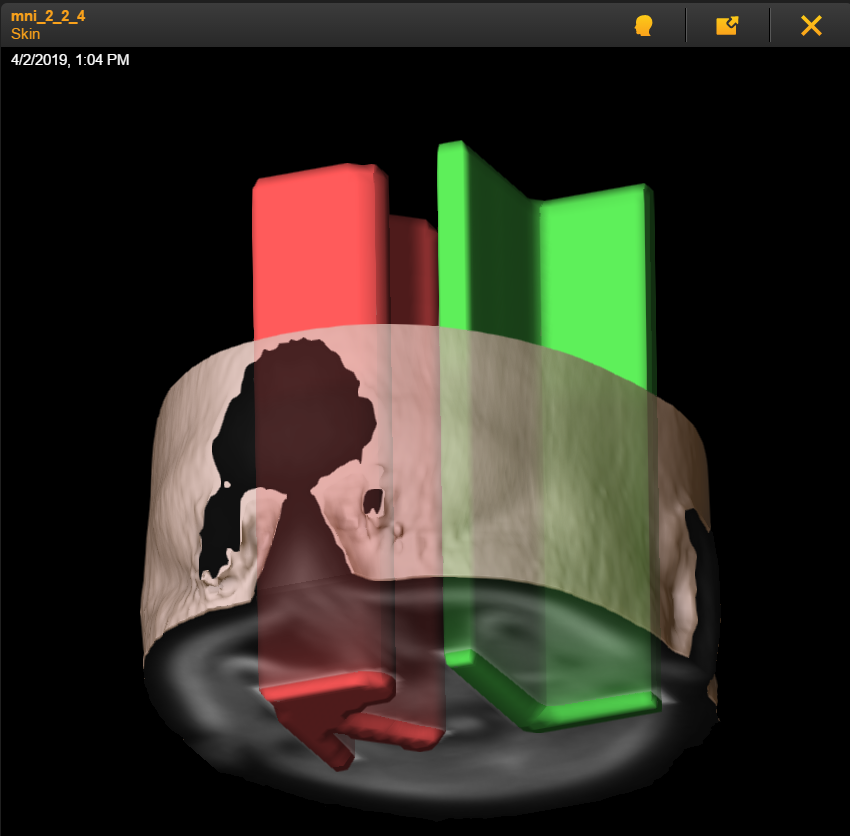


Figure 4:3D view of the dataset illustrated in Figure 3.

# References

1. Tournier J-D, Smith R, Raffelt D, Tabbara R, Dhollander T, Pietsch M, Christiaens D, Jeurissen B, Yeh C-H, Connelly A (2019) MRtrix3: A fast, flexible and open software framework for medical image processing and visualisation. NeuroImage 202:116137. https://doi.org/10.1016/j.neuroimage.2019.116137

2. Jeurissen B, Tournier J-D, Dhollander T, Connelly A, Sijbers J (2014) Multi-tissue constrained spherical deconvolution for improved analysis of multi-shell diffusion MRI data. NeuroImage 103:411–426

3. Yang JY-M, Beare R, Wu MH, Barton SM, Malpas CB, Yeh C-H, Harvey AS, Anderson V, Maixner WJ, Seal M (2019) Optic Radiation Tractography in Pediatric Brain Surgery Applications: A Reliability and Agreement Assessment of the Tractography Method. Front Neurosci 13:. https://doi.org/10.3389/fnins.2019.01254

4. Yang JY-M, Beare R, Seal ML, Harvey AS, Anderson VA, Maixner WJ (2017) A systematic evaluation of intraoperative white matter tract shift in pediatric epilepsy surgery using high-field MRI and probabilistic high angular resolution diffusion imaging tractography. J Neurosurg Pediatr 19:592–605

5. Basser PJ, Mattiello J, Lebihan D (1994) Estimation of the Effective Self-Diffusion Tensor from the NMR Spin Echo. J Magn Reson B 103:247–254. https://doi.org/10.1006/jmrb.1994.1037

6. Basser PJ, Mattiello J, LeBihan D (1994) MR diffusion tensor spectroscopy and imaging. Biophys J 66:259–267. https://doi.org/10.1016/S0006-3495(94)80775-1

7. Woolrich MW, Ripley BD, Brady M, Smith SM (2001) Temporal autocorrelation in univariate linear modeling of FMRI data. Neuroimage 14:1370–1386

8. Mason D, Scaramallion, Rhaxton, Mrbean-Bremen, Suever J, Vanessasaurus, Lemaitre G, Orfanos DP, Panchal A, Massich J, Rothberg A, Korijn Van Golen, Kerns J, Robitaille T, Shun-Shin M, Moloney, Pawelzajdel, Mattes M, Herrmann MD, Morency FC, Huicpc0207, Ferdymercury, Colonelfazackerley, Wada M, Hahn KS, Meine H, Bridge C, Bryant C, Fedorov A, Klimont A (2020) pydicom/pydicom: v1.4.1. Zenodo

9. Beare R, Lowekamp B, Yaniv Z (2018) Image Segmentation, Registration and Characterization in R with SimpleITK. J Stat Softw 86:1–35. https://doi.org/10.18637/jss.v086.i08

10. Lowekamp BC, Chen DT, Ibáñez L, Blezek D (2013) The design of SimpleITK. Front Neuroinformatics 7:45

11. Yaniv Z, Lowekamp BC, Johnson HJ, Beare R (2018) SimpleITK Image-Analysis Notebooks: a Collaborative Environment for Education and Reproducible Research. J Digit Imaging 31:290–303. https://doi.org/10.1007/s10278-017-0037-8

12. Jenkinson M, Beckmann CF, Behrens TE, Woolrich MW, Smith SM (2012) FSL. Neuroimage 62:782–790
